# Prehospital management provided by medical on-scene commanders in tunnel incidents in Oslo, Norway – an interview study

**DOI:** 10.1186/s13049-019-0649-8

**Published:** 2019-08-20

**Authors:** Johan Hylander, Britt-Inger Saveman, Ulf Björnstig, Lina Gyllencreutz

**Affiliations:** Department of Surgical and Perioperative Sciences, Surgery, Centre for Disaster Medicine, Umeå University, 901 87 Umeå, Sweden

**Keywords:** Medical on-scene commander, Prehospital emergency care, Tunnel incidents

## Abstract

**Background:**

High demands are placed on the emergency medical services to handle rescue operations in challenging environments such as tunnels. In Oslo, Norway a specialised management function within the emergency medical services, the medical on-scene commander, in line with the command structure within the police and fire brigade, might support or take over command and control from the ambulance incident officer arriving as the first ambulance personnel on scene. The aim was to shed light on the emergency medical service experiences from real tunnel incidents described by the Oslo medical on-scene commanders.

**Methods:**

Interviews were conducted with six of the seven medical on-scene commander in Oslo, Norway. Data were analysed using a qualitative content analysis.

**Results:**

The overall theme was “A need for mutual understanding of a tunnel incident”. The medical on-scene commander provided tactical support, using their special knowledge of risk objects and resources in the local area. They established operation plans with other emergency services (the police and fire brigade) in a structured and trustful way, thus creating a fluent and coordinated mission. Also, less time was spent arguing at the incident site. By socialising also outside ordinary working hours, a strong foundation of reliance was built between the different parties. A challenge in recent years has been the increasing ordinary workload, giving less opportunity for training and exchange of experiences between the three emergency services.

**Conclusions:**

The enthusiastic pioneers within the three emergency services have created a sense of familiarity and trust. A specially trained medical on-scene commander at a tunnel incident is regarded to improve the medical management. To improve efficiency, this might be worth studying for other emergency medical services with similar conditions, i.e. tunnels in densely populated areas.

## Introduction

Mass casualty incidents are a great challenge for the three emergency services (police, ambulance, and fire brigade). If an incident occurs in a confined setting such as a road or rail tunnel, the rescue operation might be challenging, especially in case of fire [1, 2]. Fires have occurred in underground structures, such at the King’s Cross station, England in 1987, where 31 people were killed [3], and in the Mont Blanc road tunnel in France that killed 39 people, including one firefighter [4, 5]. From 2011 through 2015, five large tunnel fires were reported in three road tunnels in Oslo [6]. Measures to improve survival have been taken, such as building rescue rooms and improving lighting for evacuation in heavy smoke. In addition, radio message systems warning road users on all available radio frequencies have been developed. Also signs pointing to the nearest emergency exit are regarded to be of vital importance to facilitate self-evacuation, as reported after the fire incidents in the Tauern Tunnel in Austria and the St Gotthard Tunnel in Switzerland [7, 8]. For those injured, or others who are not able to self-rescue, the complex tunnel environment puts great demands on the emergency services and their ability to handle a rescue operation [1]. Every rescue operation is, however, unique and comes with its own set of problems [9, 10].

Various structures in on-scene management are used worldwide [11]. The UK Major Incident Medical Management and Support (MIMMS) model has been an inspirational structure for the Emergency Medical Services (EMS) in Norway. MIMMS is based on the following seven components: command/control, safety (self, scene, survivor), communication, assessment, triage, treatment, and transport. Emphasis is put on inter-agency communication. Usually the initial EMS command is taken by the most experienced personnel of the first arriving ambulance – the Ambulance Incident Officer (AIO) – who is responsible for communication and coordination with other emergency services [12]. In Norway the police incident commander have the overall responsibility at an incident site [13].

In Oslo, Norway, the responsibility for EMS command and control at the incident site can be supported by a more qualified on-scene commander [13]. Due to there being 1,134 road tunnels in Norway and 11 in the Oslo area, the management of major incidents in tunnels has gained interest [6, 14]. The Oslo experiences is rather unique, which makes these of interest for exploring the medical on-scene commander function during tunnel incidents.

## Methods

### Aim

The aim of this study is to shed light on the EMS experiences from real tunnel incidents described by the Oslo medical on-scene commanders.

### Study design and context

This interview study had a qualitative design. The tunnel incident contexts, as narrated by the interviewees, were from tunnels in the Oslo region, including three fire incidents and two traffic incidents. There were no deaths in the tunnel fires, but there were fatalities in both of the traffic incidents. In the following, the term Medical on-Scene Commander (MSC) is used equivalent to the MIMMS term Medical Incident Officer. For a description of the EMS in Oslo, see Table 1. Two types of tunnels are used in the Oslo area: monotube (i.e. traffic runs bidirectionally in the same tube) and twin tube (i.e. traffic runs unidirectionally in each tube) [6, 18]. One example of an incident from the Oslofjord Tunnel is described in Table 2.

**Table 1:** The Emergency Medical System in Oslo [15–17]

Service to approx. 1.3 million inhabitantsFive regions with 15 ambulance stationsThree air ambulances, two physician-staffed ambulances, 45 emergency ambulances during the day, 29 emergency ambulances during the night 150,000 responses annually, of which 5% are joint operations with the police or fire brigade

**Table 2:** Incident description from the Oslofjord tunne [19]

A lorry caught fire 1,700 meters from the tunnel exit in the Oslofjord tunnel (7,306 m long, subsea, monotube tunnel) in June 23, 2011. The fire was detected by the Road Traffic Control Centre via road traffic cameras. Due to heavy smoke, the fire ventilation system was activated. The direction of the ventilation was predetermined, filling most of tunnel with smoke, complicating the self-rescue for the remaining road users. The tunnel had no smoke-proof evacuation rooms, and the closest exit was 3,480 meters away from the fire. The rescue operation involved about 120 personnel from the emergency services, including 57 medical personnel, 20 ambulances, 3 air ambulances, 1 on-scene commander, and 2 medical busses. A total of 37 road-users were taken to the hospital for examination. Their medical conditions were unknown, but there were no fatalities.	

### Participants and data collection

The head of the ambulance station in Oslo gave consent to the study. Oslo’s seven MSCs were contacted via email and received written information regarding the study. Six of them were interviewed. They were all male paramedics with 15 to 25 years of experience.

An interview guide consisting of semi-structured questions was used. The participants were asked to narrate a tunnel incident they had experienced and to relate their function as MSCs in supporting, or taking over, command from the paramedic AIO. The questions were divided into pre-incident, mid-incident, and post-incident. The interviews ranged from 30 to 85 min and were conducted in Norwegian and Swedish at the participants’ places of work and were recorded and transcribed verbatim.

### Data analysis

Data were analysed using qualitative content analysis [20]. To get an naïve understanding of the text, each transcribed interview was read through once, and a short summary was written [21]. Meaning units from the transcribed text relevant to the aim were extracted, and these meaning units were then condensed without losing content. The condensed units were thereafter labelled with codes [22]. Codes with similar content were clustered into subcategories that were discussed towards the interview text in the research group. After reaching consensus, nine subcategories and three categories were merged into a theme representing a “common thread” of what was latent in the text [21].

### Ethical considerations

In accordance with the Helsinki Declaration [23], the participants were informed, both orally and in writing, about the aim of the study and the possibility to decline participation and to withdraw at any time without giving a reason. Written informed consent was obtained from all of the participants. The identities of the participants cannot be uncovered based on the quotes in this study, and the interview material was coded and stored in a safe place. The respondents had no dependency on the researchers, and the study was not covered by the Swedish Act concerning the Ethical Review of Research Involving Humans (cf. [23, 24]).

## Results

Firstly, the theme “Mutual understanding of a tunnel incident” is an overall interpretation of the results. Secondly, three categories are presented together with their respective subcategories (Table 3). Quotes are used throughout to show the internal consistency.

**Table 3:** Theme, categories, and subcategories

Theme	Category	Subcategory
Mutual understanding of a tunnel incident	1. Support of EMS personnel at the incident site	Tactical leadership
		Potential safety threats
		Utilization of experience
		Implementation of knowledge
	2. Familiarity with other emergency services	Joint training efforts
		Inter-agency communication
		Feelings of trust and security
	3. Constraints and challenges in planning a rescue operation	Increased ordinary workload
		Feelings of lack of understanding and training

### Theme: mutual understanding of a tunnel incident

The theme underlines the importance of capturing a common view of the incident in all emergency services in order to optimise the rescue operations. It is important that personnel at the scene have a mutual understanding of all involved parties’ tasks and responsibilities. With a common understanding, unnecessary and time-consuming arguing might be avoided.

### Category 1: support of EMS personnel at the incident site

The MSCs described it as important to find the available resources that are needed and to keep medical personnel well informed and guided. An adequate risk assessment is also important. A wise MSC would take the medical personnel’s unique skills and experiences into account. Planning is another important task, using knowledge of geography, resources, and tunnels to formulate an optimal plan of operation.

#### Tactical leadership

The MSCs said they try to take a calm approach and a bird’s-eye view when supporting the first arriving AIO, or when taking over command. A challenge is to handle the EMS personnel’s strong will and independence, as well as expectations from the public to “do something”. A follow-up of the EMS personnel’s experiences and feelings was routine after major incidents.

*“If you are the AIO, I will be right behind you and whisper hints in your ear so you can test and improve your skills as a leader.”*

#### Potential safety threats

The MSCs expressed that EMS personnel want to help, but sometimes overlook the risks. To limit the action of medical personnel due to safety issues was a difficult question, or a “burden of command”. Overlooking risks might be fatal, and a main fear was to lose people under their command. They explained the necessity of forwarding adequate safety information from the joint command post to the EMS personnel on site, as well as to incoming medical personnel.

*“Everyone wants to help. The firefighters smell smoke, the police hear gunfire, we see blood. But someone needs to say stop until we know more about safety issues, that is the hard part.”*

The participants were of the opinion that they were the best prepared in the EMS to consider various risks at the incident site due to their more in depth knowledge of tunnel structures and existing risks. They described incidents when smoke had blinded drivers of vehicles evacuating the tunnel, endangering the lives of firefighters making their way into the tunnel. Also, firefighters had lost their orientation in thick smoke, and loud noises from tunnel ventilation fans made it difficult to hear or give orders.

*“In regard to the safety aspect of a tunnel fire we were talking about, if there is smoke in the tunnel, we do not send EMS personnel into the smoke.”*

MSCs’ exclusive knowledge also made them aware of risks of explosions in tunnels, e.g. busses powered by natural gas. Fuel leaks were described as another potential risk, especially when cutting through metal, with a risk of sparks igniting the fuel.

*“But if we are talking about gas exposure and similar things. You need to gather information, you need someone to ask the question of which cargo the lorry has, if it is dangerous.”*

#### Utilisation of experience

The MSCs said that inexperienced colleagues sometimes hesitated to enter a tunnel. They also described how recently graduated paramedics had a lack of tactical knowledge and limited experience working in tunnels. However, recently graduated colleagues were said to be eager to learn and to listen to older colleagues, which was described as a comforting factor for the MSCs. With experience comes a good “clinical eye”, i.e. the intuition to see what is needed.

*“The younger ones are more amenable to education and new knowledge. And they also look to older colleagues for support, which makes our job a lot easier.”*

#### Implementation of knowledge

MSCs’ experienced a lack of a complete tactical plan for managing tunnel incidents, and sometimes there was a need to think outside the box. Currently, they plan for access to the incident site and a suitable route for evacuating the patients, as well as for other routine factors in major incidents. Lack of personnel was, however, a concern to the MSCs. The MSCs were of the opinion that their experience of tunnel incidents might be better used by incident planners, and they would also welcome a special training course for tunnel incident management.

The MSCs experienced themselves as pioneers for a more proactive medical care because they felt it was not acceptable to just wait outside the tunnel for injured people to arrive. The MSCs had the policy that the tunnel should be “*safe enough”*, rather than entirely secured. Due to experiences from multiple fires and a deeper knowledge of the tunnel structure, a certain procedure for handling incidents has emerged.

*“So, during a rescue operation, we cannot stand outside and wait for the fire brigade to say, entirely safe. No, we have to go in.”*

### Category 2: familiarity with other emergency services

The cooperation between the three emergency services (police, ambulance, and fire brigade) and other services such as the Road Traffic Control Centre (RTCC) was described as vital for a successful rescue operation. By creating bonds also outside of working hours, a sense of trust is created, which helps all organisations work effectively together. The three emergency services also formally train together in order to improve collaboration.

#### Joint training efforts

The MSCs wished for more joint training to identify shortcomings. Training exercises in faster extraction of injured people from damaged vehicles involving the fire brigade and EMS was described as example of effective cooperation for reducing time to treatment. The importance of collaboration exercises in on-scene management was also expressed. Even if they have access to an online learning system for all three of the emergency services, they wanted a joint training centre to practice management of tunnel-related incidents.

*“I know how the tunnel is built, and I know approximately how much fire damage it can take and so on. Which means that, as an MSC from the ambulance perspective, I have the same starting point as the fire chief.”*

#### Inter-agency communication

The MSCs experienced lack of communication and sometimes conflicts between the different emergency services as a problem, which is why they have formed a special forum for sharing experiences and strategies. The MSCs also explained that in the event of a joint assignment one simple routine is that the three on-scene commanders meet and presents themselves by handshakes at the tunnel entrance. Each of the on-scene commanders is of course responsible for sharing necessary information with their units. The RTCC has the possibility to send live traffic camera images from the scene to higher command levels of the police and fire brigade, but not to the EMS, which was regarded as a shortcoming.

*“A lot of things can go wrong if we do not understand each other. We try to have a lot of focus on the same basic understanding and communication in a language that everyone can understand.”*

#### Feelings of trust and security

The MSCs had developed a habit of engaging in social activities with corresponding emergency services commanders such as dining, spending time and getting to know each other on a first-name basis. This made them more familiar with each other and built a sense of trust and an interest in helping each other.

*“So, it becomes very important to have unofficial contacts, networks, subcultures. At specific incidents and operations, unofficial contacts should also be accepted.”*

### Category 3: constraints and challenges in planning a rescue operation

MSCs described the development of having less time between calls than earlier. The drawback of this is that the time for exchanging experiences with colleagues and visiting risk locations such as tunnels has been reduced as has training together with other emergency services. In addition, the implementation of the function as MSCs in tunnel settings has been met with questions regarding productivity from higher levels.

#### Increased ordinary workload

MSCs felt that the workload, with more EMS calls and less time between calls, has increased. Earlier, the time between calls gave more time for exchanging experiences, for training, and for recuperating. This time constraint has also reduced the opportunity to share a cup of coffee with personnel from other emergency services after an incident, resulting in less time for exchanging experiences. At an incident site, MSCs also experienced an expectation to release EMS personnel at the earliest opportunity, even if they wished to have short reviews after major incidents.

*“I talked to the EMS personnel and only gave some minor feedback because most of them had to return to regular operations.”*

#### Feelings of lack of understanding and training

The MSCs felt that higher-level incident organisers have shown little interest in the MSCs’ role in major incidents. They experienced a general lack of training in tunnel settings in collaboration with other emergency services. They also felt themselves outside the planning process because they could not influence the aim of joint exercises. They also felt neglected by their superiors when coming up with new ideas for exemple utilising cancelled calls to risk objects like tunnels as training opportunities with the police and fire brigade.

It was initially difficult to get acceptance for the function of the MSC system, and it was a struggle to explain the need of an MSC in the municipality. Furthermore, they expressed a concern that when the need for an MSC arises they will be engaged in other routine tasks.

*“It happens often that a call is cancelled because we are not needed. We should use these opportunities to continue to the incident scene together with the fire brigade and police and set up the command post, and we should take 10 minutes to reflect. We should be thinking: if the incident were major, if you were the police on-scene commander in charge, what would you do? If you were the fire on-scene commander, what would you do? We would have gotten something out of it; it is the closest to a real situation we can get.”*

## Discussion

The results highlight that cooperation between the three emergency services is vital for successful rescue operations in tunnels. If there are inexperienced EMS personnel making critical decisions, or arguing between the emergency services, the operation becomes inefficient. To remove such complications, different fora to work out tactical leadership and to disseminate experiences are regarded as necessary. An MSC function, like corresponding functions in the police and fire brigade, might take over command and control for the EMS on site and/or support the AIO.

Safety threats are a major factor for all emergency services [25], and it was emphasised how important a common understanding of the safety at a tunnel incident is. The MSCs’ concept of “safe enough” pointed to a desire to advance EMS closer to the injured and to shorten time to treatment, which is a change from being completely passive outside the tunnel as in the past. However, the MSC should not neglect the risks, but make an informed decision. After the Oslo bombing in 2011, the attitude has changed to a more active role [26]. The Norwegian Directorate of Health (Helsedirektoratet) concluded in 2015 [27] that a joint decision from the command post is needed in those cases. Still, there is a debate and there is more to explore regarding EMS personnel’s’ attitudes toward safety at challenging incident sites.

The results also show that familiarity and mutual understanding between the three emergency services was achieved by joint training sessions and programs. A national course in tunnel incident management is available in Oslo, aiming for a common understanding. Also, table top exercises (as in the MIMMS course) improve the tactics for different scenarios and strengthen inter-agency collaboration. Besides table top exercises and drills, it is also important to share experiences and lessons learned [28]. However, the MSCs experienced some constraints in time, interest, and resources from management for such planning and training. Others have found similar constraints, such as budget constraints and greater demand on the ambulance services due to, for example, an increased elderly population. They also found that increased workload and poor support from the management reduces the wellbeing of the EMS personnel [29, 30]. To address this, there is a need to find innovative solutions despite the organisation’s high workload and weak upper management support.

Joint decisions based on available facts were reported to be important to avoid misunderstanding and different views of the situation. Other authors [31, 32] also found that collaboration and correct information are important, especially during the initial phase of the emergency response. In Norway, the Police Incident Officer is ultimately responsible for the work at the incident site [13]. Using a hierarchical structure as e.g. in the US, may make a joint operation effective [32]. Improvisation was regarded to be needed in special situations, and this might be more easily initiated through close cooperation.

The MSCs acknowledged the need for trust and familiarity with the other services. Due to the trust, built also outside working hours, the commanders experienced a willingness to support each other with different tasks. It was also found that trust was regarded as a factor of success, but needed to be developed before collaborating in the field. Such workplace culture promoted positive group dynamics [33]. Beside trust between the emergency services, MSCs might also place trust in the less experienced first-arriving AIOs, helping them grow in their leadership role. Others [34] also found that this type of support is an important factor for personal development.

### Methodological considerations

This study is based on interviews with six out of seven possible interviewees, which is a limited, but nearly a complete material. The interviews were thick with rich descriptions, and the analysis of the texts gave results that are considered valid and following the regimen of qualitative content analysis [20]. Consensus was reached for subcategories and categories by discussion among the authors, and being familiar with EMS care, as the authors are, might have minimised the risk for misunderstandings.

## Conclusion

The MSC function in Oslo was experienced to be a valuable improvement in the EMS quality and efficiency, especially in challenging contexts such as tunnel incidents. These results might be of interest for EMS in similar contexts of underground incidents in densely populated areas.

## Data Availability

The source material used in the present study is available from the corresponding author upon reasonable request.

## Abbreviations

AIO: Ambulance Incident Officer
EMS: Emergency Medical Services
MCS: Medical on-Scene Commander
MIMMS: Major Incident Medical Management and Support
RTCC: Road Traffic Control Centre
